# The sociocognitive processes of ideological obsession: review and policy implications

**DOI:** 10.1098/rstb.2020.0144

**Published:** 2021-04-12

**Authors:** Jocelyn J. Bélanger

**Affiliations:** Department of Psychology, New York University Abu Dhabi, Saadiyat Island, PO Box 129188, Abu Dhabi, United Arab Emirates

**Keywords:** ideological obsession, passion, radicalization, violent extremism

## Abstract

Understanding what motivates people to join violent ideological groups and engage in acts of cruelty against others is of great social and societal importance. In this paper, I posit that one necessary element is ‘ideological obsession’—an ideological commitment fuelled by unmet psychological needs and regulated by inhibitory and ego-defensive mechanisms. Drawing from evidence collected across cultures and ideologies, I describe four processes through which ideological obsession puts individuals on a path towards violence. First, ideological obsession deactivates moral self-regulatory processes, allowing unethical behaviours to be carried out without self-recrimination. Second, ideologically obsessed individuals are easily threatened by information that criticises their ideology, which in turn leads to hatred and violent retaliation. Third, ideological obsession changes people's social interactions by making them gravitate towards like-minded individuals who support ideological violence. As these social networks become more interconnected, they amplify one's adherence to violent extremism. Finally, ideologically obsessed individuals are prone to psychological reactance, making them immune to communication strategies intended to dissuade them from using violence. In fact, messages espousing non-violence can have the opposite effect by reinforcing their violence-supporting ideology. I conclude by presenting evidence-based strategies to prevent radicalisation leading to violence for individuals in pre-criminal spaces.

This article is part of the theme issue ‘The political brain: neurocognitive and computational mechanisms’.

## 1. ‘Needs frustration’ as a source of addiction and obsession

Popularly understood, addiction is a global dilemma. Worldwide, 1.3 billion people are addicted to smoking, 240 million people are dependent on alcohol and 15 million people are reliant on intravenous drugs [1]. Common definitions of addiction also extend to obsessive eating (650 million people are overweight), Internet pornography (13% of Internet traffic), gambling (about 1.5% of the global adult population) and oniomania (compulsive buying habits, which are present in 1–6% of the general population) [2–5].

However, there is another set of destructive behaviours that are rarely subsumed under the umbrella of addictions: the phenomenon of ideological obsession, also known as obsessive ideological passion, which has been defined as the overwhelming engagement in a political or religious ideology [6–8]. Much like other addictions [9], the manifestations of ideological obsession consist of strong irresistible impulses, recurrent conflicts with other life domains, giving up other activities and the pursuit of one's ideology despite it being both psychologically and physically hazardous [6–8]. Ideological obsession can be reliably measured with the obsessive passion scale, a psychometric instrument with a 7-point Likert scale that can be adapted to any political or religious ideology [6–8]. There are different degrees of ideological obsession. Therefore, there is no cut-off point on the scale and ideological obsession is treated as a continuous variable. The more people agree with its statements (e.g. ‘my ideology is the only thing that I can think of’ and ‘my ideology is so exciting that I sometimes lose control over it’), the more they tend to support violent extremism. The purpose of this paper is to illuminate this concept of addiction and review studies that have been undertaken to comprehend the sociocognitive mechanisms that connect ideological obsession to violent extremism.

To guide this analysis, we must examine what addictions have in common. One of the first commonalities of addictions is their self-defeating nature, meaning that addictions serve a goal while undermining other pursuits. Psychologists refer to such behaviours as counterfinal means [10,11]. These are ways of comporting oneself that promise great rewards, but also come with a hefty price tag.

Consider drinking alcohol: consumption is a means that serves a goal (such as coping with negative affectivity), with potentially harmful health consequences. The same rationale could be applied to political activists during a protest. Political violence (e.g. throwing petrol bombs at the police) might provide activists with a sense that they are making headway towards achieving their political ends, but the cost might be years in jail, or worse, a death sentence. These actions are taken voluntarily. In fact, motivation science indicates that the greater the perceived cost associated with counterfinal means, the more people perceived them as instrumental to the goal they purportedly serve. That is because people generally believe in a ‘no pain, no gain’ heuristic that becomes especially salient when reaching one's goal seems unlikely [10,11].

What this shows is that addictions are goal-driven. Far from being a trifling observation, this formulation implies that, although self-defeating, addictions are self-regulatory successes (one sets a goal and achieves it) rather than failures, an idea that runs counter to the widespread assumption that addictions reflect an inability to achieve goals owing to poor self-control [12–15].

But if addictions are indeed goal-driven, what are people trying to achieve through profound identity transformations that can cause great harm to themselves and others? Claiming that addictions serve only one goal would be an oversimplification, but there is substantial empirical evidence suggesting that addictions share a common genesis: the desire to fill a void. In fact, whether people indulge excessively in drug use or become firebrands of an ideology, addictions often originate from people experiencing a feeling that their own lives are worthless, spoiled and meaningless.

My colleagues and I refer to this phenomenon as the loss of personal significance [16–19], but psychologists have evoked a similar psychological malaise with different terminologies such as ‘dislocation’, ‘lack of need satisfaction’ and ‘poverty of the spirit’ [20–22]. Interestingly, irrespective of the ‘ism’ for which people are willing to risk life and limb (be it jihadism, ethnonationalism or environmentalism), our findings indicate that people harbouring radical ideas are generally afflicted by this aversive psychological state [23–25]. They believe that their sacrifice or act of ‘martyrdom’ will serve their group survival, provide them with a hero status and enshrine them forever in the collective memory of their group. This represents the pinnacle of personal significance and is perhaps one of the oldest narratives used by propagandists to produce ideologues marching in lockstep to the drumbeat of extremism.

Remarkably, even rats, which communicate among each other using high-frequency sounds, are prone to developing addictions when their social needs are not met. While rats do not engage in kamikaze behaviours like other species (such as some worker ants, honeybees and wasps [26–28]), they do exhibit self-destructive tendencies. For instance, when rats are housed in enriched environments known as ‘rat parks’, which provide room to roam, play, socialize and mate, they are less likely to self-administer morphine than rats isolated in a standard laboratory cage [29]. Again, this shows that addictions have a self-regulatory purpose, in that they are attempts to restore fundamental needs, especially when the environment does not provide viable routes to satisfaction. From this standpoint, addictions in general, and ideological obsessions in particular, are coping mechanisms, a latent potential that expresses itself under particular social circumstances. An important corollary is that ideological obsession is a psychological process whereby individuals progressively become obsequious servants to an ideology. Let us examine how this process unfolds.

## 2. Sociocognitive mechanisms of ideological obsession

**Figure 1.**
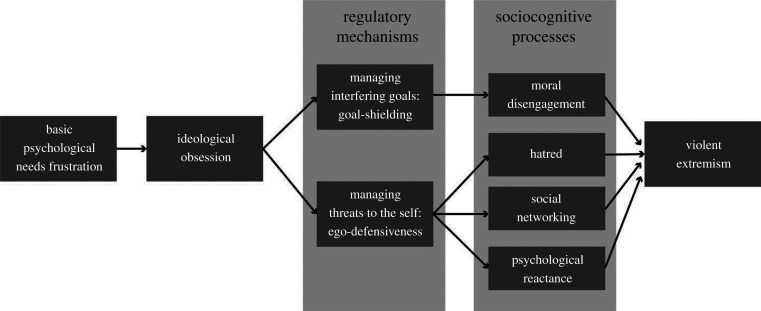
The theory of ideological obsession: from needs frustration to violent extremism.

The psychological transformation that characterizes an individual's ideological obsession begins with the chronic frustration of basic psychological needs (figure 1). This can happen because of a person's life circumstances (e.g. a personal failure) or because one's dignity is continuously denied by the prevailing economic, social and political order. The person's grievance can take many forms (inequality, marginalization, disempowerment), but is inevitably associated with a state of distress and humiliation.

The constant frustration of basic needs has profound psychological implications. Social pain, neuroscience suggests, affects the same brain regions as physical pain [30]. And when people are confronted with such torment, they will do about anything to return to a state of homeostasis [31].

That said, people do not necessarily turn to violence because their needs are chronically frustrated; individuals can be extraordinarily resilient to adversity. Usually, they display high ambition and find significance in various constructive non-violent ways; they seek to achieve social status (e.g. athlete, physician, engineer) and become people of standing in their community [16]. However, if these strategies are perceived as unavailable, unattainable or unlikely to redress one's significance, then people are either crushed by hopelessness or tempted to use ideological violence as a strategy of last resort to provoke radical societal changes [32,33]. If nothing else, commitment to an ideology is a means for individuals to attain—at least in their minds, if not also among their peers—a desired social status that might otherwise be unavailable to them.

The issue, however, is that when people are convinced that ideological violence is the only means of addressing their grievance, they become overly involved in the need-satisfying ideology [34], completely absorbed by their political engagement. Over time, they also become possessed by a strong urge to engage in political or religious activism and their self-worth inextricably contingent on the progression of their ideology [8,35].

There are two important shifts in self-regulation associated with this psychological transformation: (i) the reliance on goal-shielding to manage goals that interfere with one's ideological engagement, and (ii) ego-defensive reactions to manage threats to the self. Each of these regulatory mechanisms is associated with different sociocognitive processes contributing to violent extremism. These mechanisms and processes are not unique to violent extremists; they can be experienced by any human being. However, the present theoretical framework posits that it is their simultaneous manifestation in a single individual owing to ideological obsession that makes them a particularly dangerous, combustible, mixture.

### (a) Goal-shielding

Because ideologically obsessed individuals are completely absorbed by their beliefs, conflicts with other life domains—such as family, education and work—more easily arise [36]. These conflicts are frustrating in that they divert attentional resources from the need-satisfying ideology. To manage the chronic experience of goal-conflict, individuals engage in goal-shielding, a mechanism that ‘automatically regulates one's attentional focus by inhibiting potentially distracting alternative goals’ [37]. This means that when ideologically obsessed individuals are exposed to goals unrelated to the pursuit of their ideological engagement, other goals are instantly minimized, suppressed and forgotten [38].

The suppression of alternative goals plays an important role in connecting ideological obsession to violent extremism. Suppressed goals cannot vie for attention and can, therefore, be neglected. When this happens, the ideological pursuit becomes untrammelled by constraints and individuals develop a preference for counterfinal means that serve their ideology and undermine other goals [39]. In other words, when the end justifies the means, there are few safeguards left to prevent people from engaging in self-defeating behaviours. One last line of defence might be the moral self-sanctions that constrain the repertoire of acceptable behaviour. However, there is evidence that ideological obsessions also suppress these considerations, leading to moral disengagement [40], the dehumanization of outgroup members, and the use of violence without self-recrimination, even appearing righteous to its perpetrators [41,42].

In a study investigating this phenomenon [40], my colleagues and I instructed self-identified environmentalists to look at the faces of outgroup members (such as people who support the oil industry) displayed on a computer screen. Using specialized software, the facial stimuli progressively morphed into objects such as statues and dolls. Participants were instructed to hit the space bar when they believed the faces no longer looked human. The results showed that the greater the ideological obsession, the quicker environmentalists were at making such judgements, indicating that they swiftly dehumanized outgroup members. Importantly, the speed at which they made such decisions predicted their support for ideological violence, such that faster judgements were related to greater support for violence against outgroup members.

### (b) Ego-defensiveness

The second regulatory mechanism that promotes violent extremism is ego-defensiveness. When people's self-worth revolves exclusively around their ideological engagement, their identity becomes progressively unidimensional and psychologically impoverished, with fewer interests [43]. The fact that their sense of identity becomes fragile and uncertain has several consequences.

First, ideologically obsessed individuals are more easily threatened by information challenging their belief system, producing hatred and a desire to retaliate excessively. In an interesting demonstration of this phenomenon, Rip *et al*. [6] had Muslims read Pope Benedict XVI's Regensburg address, in which he quoted a fourteenth-century Christian emperor who stated that Islam is associated with ‘things only evil and inhuman’. The study's authors found that ideologically obsessed individuals who were exposed to the Pope's speech felt more hatred than those who were not exposed, which in turn predicted individual support to publicly punish those ‘who dare offend Islam’ by ‘respond[ing] with weapons and prepar[ing] for a holy war’.

Second, in addition to laying the foundation for developing an animus towards those who contradict one's viewpoint, having a fragile ego influences the way in which people interact socially. In a recent set of studies [44], my colleagues and I made the prediction that ideologically obsessed individuals would be motivated to anchor themselves in groups with radical ideologies. Why? Because self-doubt riddles people's mind with considerable anxiety and radical groups are the perfect medicine for those suffering from this ailment. Indeed, radical groups are particularly effective in providing a clear sense of purpose and identity to their members [45]. Their simplistic, rigid and absolutist narratives provide clearly delineated group boundaries: ‘you are either with us or against us’. They also tend to be highly cohesive hierarchical structures under the undisputed control of a leader [46] who severely punishes dissent, thereby creating conformity pressure and clarity regarding how one should think or act [47].

Supporting our predictions, we found that ideologically obsessed individuals in Spain (associated with both right- and left-leaning movements) and Muslims in Pakistan were more likely to befriend individuals who support ideological violence, which in turn predicted their own willingness to engage in similar actions. We also found that study participants positively evaluated the social media profiles of strangers devoted to a similar ideology, but only when those profiles were supportive of radical activities and less when they were in favour of peaceful activism. These experimental results show that individuals with little perceived personal significance are vulnerable to the draw of radical groups that dispel uncertainty with narratives that justify aggression against the outgroup.

In addition to these principles, we examined whether certain network structures increase affiliation with radical groups. Drawing from social network analysis, we examined the concept of network density—measured by the proportion of possible social ties in a network that are actually present—representing the extent to which members in a social network are connected to one another (known as network cohesion) [48]. Groups with greater network density tend to be highly entitative social units that provide a sense of community and ‘we-ness’ to members [49–52]. Our results indicate that the relationships between ideological obsession, affiliation to a radical group and support for violence are magnified when individuals are embedded in dense social networks where group members are highly interconnected.

## 3. Restoring balance

How can we curtail the phenomenon of ideological obsession and perhaps even reverse it? One commonly held theory among governments, practitioners and non-governmental organizations is that individuals radicalize because they have been seduced by a twisted, fact-distorting, ideology. Based on that thesis, challenging extremist narratives, either online or offline, consists of denouncing these perverted ideas with credible community voices that undercut recruitment into radical organizations. For the past 2 decades or so, this approach has been spearheaded by many agencies, and yet, until recently, not a single piece of data supporting its effectiveness had been produced.

In an experiment designed to attenuate the appeal of ISIS among American Muslims [53], my colleagues and I crafted different counter-narratives to persuade individuals not to join this terrorist organization. Contrary to the notion that challenging extremist narratives would break the ideological spell, we found that those at a greater risk of ideological obsession reported more, not less, support for ISIS! We also showed similar results in experiments with a wider range of ideologies—including environmentalism, the ‘Black Lives Matter’ movement, the US Republican Party and pro-life supporters [54]. This is a classic case of psychological reactance: persuasive appeals produce the exact opposite behaviour because they threaten people's freedom to act as desired. Ideologically obsessed individuals are prone to psychological reactance because their sense of worth derives almost exclusively from the pursuit of their ideology. Therefore, threatening their belief system is counterproductive, unless their ego-defensive mechanisms have been preemptively defused. Supporting that hypothesis, we found that counter-narratives can mitigate support for ideological violence, but only when people can assert their personal significance by expressing their core values (a technique known as self-affirmation [55]), otherwise counter-narratives tend to backfire.

Given these findings, it appears that momentarily finding personal significance can reduce ideological obsession and lessen one's support for violence, but what about longer-term strategies? In another recent study [56], we aimed to reduce people's obsessive passion for an activity (such as exercising) by helping them design a daily routine in which they could incorporate new goals in the pursuit of their obsessive passion (exercising with friends, for instance)—echoing the rat park study mentioned previously [29]. For seven consecutive days, participants acted on their plan, thereby enriching their lives with new experiences. Two weeks after the intervention, we followed up with survey subjects and found that their obsession for an activity had decreased and they were in greater control of their lives. This effect was explained by increased needs satisfaction and decreased needs frustration.

Overall, the results suggest that helping individuals diversify their repertoire of activities can reduce compensatory behaviours geared towards satisfying their fundamental needs. Although these findings were not in the context of ideological obsession *per se*, they are a proof of concept, paving the way for future prevention strategies designed to steer individuals away from violent extremism.

### Coda

Radicalization is an addiction to an ideology; it is an obsession to a belief system stoked by the loss of personal significance that triggers a set of sociocognitive mechanisms leaving individuals prone to engaging in ideological violence. Ideologically obsessed individuals are ego-defensive and easily threatened by information challenging their belief system. Their ideological obsession chronically conflicts with other life domains, which in turn produces goal-shielding and facilitates the use of self-defeating, counterfinal behaviours and the dehumanization of outgroup members. These processes are accelerated by people joining networks of like-minded individuals who support ideological violence and provide a sense of camaraderie and meaning. Reversing the radicalization process involves restoring people's sense of personal significance through better self-regulatory strategies to attain a richer, more satisfying and better-balanced life.

## Data accessibility

This article has no additional data.

## References

[1] Gowing LR, Ali RL, Allsop S, Marsden J, Turf EE, West R, Witton J. 2015 Global statistics on addictive behaviours: 2014 status report. Addiction **110**, 904-919. (10.1111/add.12899) 25963869

[2] World Health Organization. Obesity and overweight [Internet]. See https://www.who.int/news-room/fact-sheets/detail/obesity-and-overweight [cited 22 July 2020]

[3] Ogas O, Gaddam S. 2011 A billion wicked thoughts: what the internet tells us about sexual relationships. New York, NY: Plume.

[4] Abbott M. 2017 The epidemiology and impact of gambling disorder and other gambling-related harm. In Discussion paper for the 2017 WHO forum on alcohol, drugs and addictive behaviours, pp. 26-28. Geneva, Switzerland: WHO Headquarters.

[5] Pazarlis P, Katsigiannopoulos K, Papazisis G, Bolimou S, Garyfallos G. 2008 Compulsive buying: a review. Ann. Gen. Psychiatry **7**(S1), S273. (10.1186/1744-859X-7-S1-S273)

[6] Rip B, Vallerand RJ, Lafrenière MA. 2012 Passion for a cause, passion for a creed: on ideological passion, identity threat, and extremism. J. Pers. **80**, 573-602. (10.1111/j.1467-6494.2011.00743.x) 22091560

[7] Gousse-Lessard AS, Vallerand RJ, Carbonneau N, Lafrenière MAK. 2013 The role of passion in mainstream and radical behaviors: a look at environmental activism. J. Environ. Psychol. **35**, 18-29. (10.1016/j.jenvp.2013.03.003)

[8] St-Louis AC, Carbonneau N, Vallerand RJ. 2016 Passion for a cause: how it affects health and subjective well-being. J. Pers. **84**, 263-276. (10.1111/jopy.12157) 25546175

[9] American Psychiatric Association. 2013 Diagnostic and statistical manual of mental disorders, 5th edition. Arlington, VA: American Psychiatric Association.

[10] Bélanger JJ, Schumpe BM, Lafrenière MA, Giacomantonio M, Brizi A, Kruglanski AW. 2016 Beyond goal commitment: how expectancy shapes means evaluation. Motiv. Sci. **2**, 67. (10.1037/mot0000031)

[11] Schumpe BM, Bélanger JJ, Dugas M, Erb HP, Kruglanski AW. 2018 Counterfinality: on the increased perceived instrumentality of means to a goal. Front. Psychol. **9**, 1052. (10.3389/fpsyg.2018.01052) 30022959 PMC6040204

[12] Baumeister RF, Heatherton TF. 1996 Self-regulation failure: an overview. Psychol. Inquiry **7**, 1-5. (10.1207/s15327965pli0701_1)

[13] Kahneman D, Slovic SP, Slovic P, Tversky A. 1982 Judgment under uncertainty: heuristics and biases. Cambridge, UK: Cambridge University Press.

[14] Wagner DD, Heatherton TF. 2015 Self-regulation and its failure: the seven deadly threats to self-regulation. In APA handbook of personality and social psychology, volume 1: attitudes and social cognition (eds E Borgida, J Bargh), pp. 805-842. Washington, DC: American Psychological Association.

[15] Hart CL, Marvin CB, Silver R, Smith EE. 2012 Is cognitive functioning impaired in methamphetamine users? A critical review. Neuropsychopharmacology. **37**, 586-608. (10.1038/npp.2011.276) 22089317 PMC3260986

[16] Kruglanski AW, Bélanger JJ, Gunaratna R. 2019 The three pillars of radicalization: needs, narratives, and networks. New York, NY: Oxford University Press.

[17] Dugas M, Bélanger JJ, Moyano M, Schumpe BM, Kruglanski AW, Gelfand MJ, Touchton-Leonard K, Nociti N. 2016 The quest for significance motivates self-sacrifice. Motiv. Sci. **2**, 15. (10.1037/mot0000030)

[18] Bélanger JJ. 2017 The rise and fall of violent extremism: the science behind community-based interventions. In The motivation–cognition interface (eds CE Kopetz, A Fishbach), pp. 170-195. New York, NY: Routledge.

[19] Schumpe BM, Bélanger JJ, Moyano M, Nisa CF. 2018 The role of sensation seeking in political violence: an extension of the significance quest theory. J. Pers. Soc. Psychol. **118**, 743-761. (10.1037/pspp0000223) 30382738

[20] Polanyi K, MacIver RM. 1944 The great transformation. Boston, MA: Beacon Press.

[21] Ryan RM, Deci EL. 2017 Self-determination theory: basic psychological needs in motivation, development, and wellness. New York, NY: Guilford Press.

[22] Alexander B. 2010 The globalization of addiction: a study in poverty of the spirit. Oxford, UK: Oxford University Press.

[23] Webber D et al. 2018 The road to extremism: field and experimental evidence that significance loss-induced need for closure fosters radicalization. J. Pers. Soc. Psychol. **114**, 270. (10.1037/pspi0000111) 28872332

[24] Webber D, Chernikova M, Kruglanski AW, Gelfand MJ, Hettiarachchi M, Gunaratna R, Lafreniere MA, Belanger JJ. 2018 Deradicalizing detained terrorists. Pol. Psychol. **39**, 539-556. (10.1111/pops.12428)

[25] Bélanger JJ, Moyano M, Muhammad H, Richardson L, Lafrenière MA, McCaffery P, Framand K, Nociti N. 2019 Radicalization leading to violence: a test of the 3N model. Front. Psychiatry **10**, 42. (10.3389/fpsyt.2019.00042) 30853917 PMC6396731

[26] Bélanger JJ, Schumpe BM, Menon B, Conde J, Nociti N. 2018 Self-sacrifice for a cause: a review and an integrative model. In The SAGE handbook of personality and individual differences: origins of personality and individual differences (eds V Zeigler-Hill, TK Shackelford), pp. 465-485. Thousand Oaks, CA: SAGE.

[27] Davidson DW, Salim KA, Billen J. 2012 Histology of structures used in territorial combat by Borneo's ‘exploding ants’. Acta Zool. **93**, 487-491. (10.1111/j.1463-6395.2011.00523.x)

[28] Jones TH, Clark DA, Edwards AA, Davidson DW, Spande TF, Snelling RR. 2004 The chemistry of exploding ants, *Camponotus* SPP. (*Cylindricus* COMPLEX). J. Chem. Ecol. **30**, 1479-1492. (10.1023/B:JOEC.0000042063.01424.28) 15537154

[29] Alexander BK, Coambs RB, Hadaway PF. 1978 The effect of housing and gender on morphine self-administration in rats. Psychopharmacology (Berl.) **58**, 175-179. (10.1007/BF00426903) 98787

[30] Eisenberger NI. 2003 Does rejection hurt? An fMRI study of social exclusion. Science **302**, 290-292. (10.1126/science.1089134) 14551436

[31] Kruglanski AW, Gelfand MJ, Bélanger JJ, Sheveland A, Hetiarachchi M, Gunaratna R. 2014 The psychology of radicalization and deradicalization: how significance quest impacts violent extremism. Pol. Psychol. **35**, 69-93. (https://doi:10.1111/pops.12163)

[32] Drury J, Reicher S. 2005 Explaining enduring empowerment: a comparative study of collective action and psychological outcomes. Eur. J. Soc. Psychol. **35**, 35-58. (10.1002/ejsp.231)

[33] Obaidi M, Bergh R, Akrami N, Anjum G. 2019 Group-based relative deprivation explains endorsement of extremism among Western-born Muslims. Psychol. Sci. **30**, 596-605. (10.1177/0956797619834879) 30875267

[34] Lalande D, Vallerand RJ, Lafrenière MA, Verner-Filion J, Laurent FA, Forest J, Paquet Y. 2017 Obsessive passion: a compensatory response to unsatisfied needs. J. Pers. **85**, 163-178. (10.1111/jopy.12229) 26385633

[35] Mageau GA, Carpentier J, Vallerand RJ. 2011 The role of self-esteem contingencies in the distinction between obsessive and harmonious passion. Eur. J. Soc. Psychol. **41**, 720-729. (10.1002/ejsp.798)

[36] Séguin-Lévesque C, Laliberté ML, Pelletier LG, Blanchard C, Vallerand RJ. 2003 Harmonious and obsessive passion for the Internet: their associations with the couple's relationship. J. Appl. Soc. Psychol. **33**, 197-221. (10.1111/j.1559-1816.2003.tb02079.x)

[37] Shah JY, Friedman R, Kruglanski AW. 2002 Forgetting all else: on the antecedents and consequences of goal shielding. J. Pers. Soc. Psychol. **83**, 1261. (10.1037/0022-3514.83.6.1261) 12500810

[38] Bélanger JJ, Lafrenière MA, Vallerand RJ, Kruglanski AW. 2013 When passion makes the heart grow colder: the role of passion in alternative goal suppression. J. Pers. Soc. Psychol. **104**, 126. (10.1037/a0029679) 22905768

[39] Bélanger JJ, Schumpe BM, Nisa CF. 2019 How passionate individuals regulate their activity with other life domains: a goal-systemic perspective. J. Pers. **87**, 1136-1150. (10.1111/jopy.12463) 30742310

[40] Bélanger JJ, Schumpe BM, Nociti N, Moyano M, Dandeneau S, Chamberland PE, Vallerand RJ. 2019 Passion and moral disengagement: different pathways to political activism. J. Pers. **87**, 1234-1249. (10.1111/jopy.12470) 30802958

[41] Bandura A. 1999 Moral disengagement in the perpetration of inhumanities. Pers. Soc. Psychol. Rev. **3**, 193-209. (10.1207/s15327957pspr0303_3) 15661671

[42] Fiske AP, Rai TS. 2015 Virtuous violence: hurting and killing to create, sustain, end, and honor social relationships, p. 42. Cambridge, UK: Cambridge University Press.

[43] Bélanger JJ, Lafrenière MA, Vallerand RJ, Kruglanski AW. 2013 Driven by fear: the effect of success and failure information on passionate individuals' performance. J. Pers. Soc. Psychol. **104**, 180. (10.1037/a0029585) 22889073

[44] Bélanger JJ et al. Supporting political violence: the role of ideological passion and social network. Group Process Intergroup Relat. **23**, 1187-1203. (10.1177/1368430220933954).

[45] Hogg MA, Sherman DK, Dierselhuis J, Maitner AT, Moffitt G. 2007 Uncertainty, entitativity, and group identification. J. Exp. Soc. Psychol. **43**, 135-142. (10.1016/j.jesp.2005.12.008)

[46] Milla MN, Hudiyana J, Cahyono W, Muluk H. 2020 Is the role of ideologists central in terrorist networks? A social network analysis of Indonesian terrorist groups. Front. Psychol. **11**, 333. (10.3389/fpsyg.2020.00333) 32194482 PMC7063091

[47] Della Porta D. 2013 Clandestine political violence. New York, NY: Cambridge University Press.

[48] De Nooy W, Mrvar A, Batagelj V. 2018 Exploratory social network analysis with Pajek: revised and expanded edition for updated software. Cambridge, UK: Cambridge University Press.

[49] Igarashi T, Kashima Y. 2011 Perceived entitativity of social networks. J. Exp. Soc. Psychol. **47**, 1048-1058. (10.1016/j.jesp.2011.04.008)

[50] Sohn D. 2014 Coping with information in social media: the effects of network structure and knowledge on perception of information value. Comput. Hum. Behav. **32**, 145-151. (10.1016/j.chb.2013.12.006)

[51] Brown R, Wade G. 1987 Superordinate goals and intergroup behaviour: the effect of role ambiguity and status on intergroup attitudes and task performance. Eur. J. Soc. Psychol. **17**, 131-142. (10.1002/ejsp.2420170202)

[52] Podolny JM, Baron JN. 1997 Resources and relationships: social networks and mobility in the workplace. Am. Sociol. Rev. **62**, 673-693. (10.2307/2657354)

[53] Bélanger JJ, Nisa CF, Schumpe BM, Gurmu T, Williams MJ, Putra IE. 2020 Do counter-narratives reduce support for ISIS? Yes, but not for their target audience. Front. Psychol. **11**, 1059. (10.3389/fpsyg.2020.01059) 32655429 PMC7325943

[54] Bélanger JJ, Schumpe BM, Nisa CF, Moyano M. 2020 When counter messaging backfires: obsessive passion as a predictor of reactance. Motiv. Sci. (10.1037/mot0000206)

[55] Steele CM. 1988 The psychology of self-affirmation: sustaining the integrity of the self. Adv. Exp. Soc. Psychol. **21**, 261-302.

[56] Bélanger JJ, Nisa CF, Schumpe BM, Chamberland P-E. 2019 Using implementation intentions to change passion: the role of environmental mastery and basic psychological needs. Motiv. Sci. **5**, 343-356. (10.1037/mot0000125)

